# Potholes and traffic signs detection by classifier with vision transformers

**DOI:** 10.1038/s41598-024-52426-4

**Published:** 2024-01-26

**Authors:** Satish Kumar Satti, Goluguri N. V. Rajareddy, Kaushik Mishra, Amir H. Gandomi

**Affiliations:** 1 Department of Computer Science and Engineering, VFSTR Deemed to be University, Guntur, 522213 India; 2 Department of Computer Science and Engineering, GITAM Deemed to be University, Visakhapatnam, 530045 India; 3 Faculty of Engineering and Information Technology, University of Technology Sydney, Ultimo, NSW 2007 Australia; 4 University Research and Innovation Center (EKIK), Óbuda University, 1034 Budapest, Hungary

**Keywords:** Civil engineering, Computational science, Mathematics and computing

## Abstract

Detecting potholes and traffic signs is crucial for driver assistance systems and autonomous vehicles, emphasizing real-time and accurate recognition. In India, approximately 2500 fatalities occur annually due to accidents linked to hidden potholes and overlooked traffic signs. Existing methods often overlook water-filled and illuminated potholes, as well as those shaded by trees. Additionally, they neglect the perspective and illuminated (nighttime) traffic signs. To address these challenges, this study introduces a novel approach employing a cascade classifier along with a vision transformer. A cascade classifier identifies patterns associated with these elements, and Vision Transformers conducts detailed analysis and classification. The proposed approach undergoes training and evaluation on ICTS, GTSRDB, KAGGLE, and CCSAD datasets. Model performance is assessed using precision, recall, and mean Average Precision (mAP) metrics. Compared to state-of-the-art techniques like YOLOv3, YOLOv4, Faster RCNN, and SSD, the method achieves impressive recognition with a mAP of 97.14% for traffic sign detection and 98.27% for pothole detection.

## Introduction

In the present context, global transportation options encompass air travel, metros, buses, and various personal vehicles. Among these, road transportation stands out as a widespread and economical means of connecting diverse locations. However, owing to diverse road conditions or lapses in driver attention, accidents are a daily occurrence. While drivers are expected to focus on the road, additional assistance can enhance their awareness and alert them to potential emerging hazards. This assistance has the potential to minimize human errors by actively monitoring the driving environment and providing timely warnings along with recommendations and alarms. This study focuses on the development of an Intelligent Transport System designed to alert drivers to potential degradation. Specifically, the research addresses the challenges associated with detecting potholes and traffic signs in the conditions prevalent on Indian roads.

The statistics starkly illustrate the detrimental impact of potholes on road safety. In 2018, accidents stemming from potholes led to the tragic loss of 15 lives. The gravity of the situation is further highlighted by the figures for preceding years, with 9423 accidents and 3597 fatalities in 2017, 6424 accidents and 2324 lives lost in 2016, and 10,876 incidents resulting in 3416 deaths in 2015^1^. Similarly, in 2014, accidents related to potholes claimed the lives of 3039 individuals. According to the Ministry of India, Uttar Pradesh recorded the highest number of fatalities attributed to potholes in 2018^2^, with 1043 cases, followed by Haryana with 222 instances and Maharashtra with 166 fatalities. The issue persisted, causing 4775 incidents in 2019 and 3564 accidents in 2020. This worrisome trend highlights the pressing need for effective measures to address the impact of potholes on road safety in India.

The efficacy of Intelligent Transport Systems, as well as autonomous and assisted driving, hinges heavily on the precise identification of traffic signs and potholes^3^. This identification empowers drivers with real-time information through automated traffic sign detection and pothole detection, enabling better control of their actions and elevating the safety and convenience of operating motor vehicles. Automated traffic sign and pothole detection is a fundamental component for automated driving systems. Its potential advantages for the future are substantial. Nonetheless, challenges such as variations in illumination, adverse weather conditions contribute to the intricacies of real-world traffic scenarios, indicating that the domain of traffic sign and pothole detection still harbors numerous unanswered questions. The crucial aspect of traffic sign and pothole detection lies in the ability to recognize minor signs and tiny potholes within a complex environment, ensuring the resilience and accuracy of the detection system. Numerous studies have extensively explored methods for identifying traffic signs^4^. Leveraging the geometric shapes and vibrant colors of traffic signs, algorithms based on color and shape have been proposed. These algorithms extract relevant information to produce features from the region of interest (ROI) containing the traffic signs.

In recent times, deep learning has gained popularity in the realm of traffic sign and pothole detection. Certain attention-based detection approaches incorporate an attention module to extract the ROI from the input image and optimize features against complex backgrounds^5^. The integration of these two techniques significantly improves the accuracy of recognizing minor traffic signs while reducing false alarms. However, the deployment of deep neural networks on movable platforms poses challenges due to time-consuming processes and high computational requirements. Detecting traffic signs and potholes on moving devices becomes challenging. Consequently, many approaches opt for lightweight network models developed through compression techniques. This approach aims to reduce the computational load, enabling real-time traffic sign identification on mobile platforms.

Improving the robustness and accuracy of traffic sign recognition and pothole detection systems relies heavily on the ability to effectively recognize and interpret minor traffic signs and tiny potholes within complicated different environments. Extensive research has been devoted to the exploration of various techniques in this domain^4^. Given that traffic signs typically exhibit geometric shapes such as triangles, circles, and rectangles, coupled with vibrant colors, several algorithms have been proposed that leverage color and shape-based information for traffic sign detection. These proposed algorithms extract pertinent features from the region of interest (ROI) encompassing the traffic sign. In recent years, deep learning has gained significant popularity within the realm of traffic sign detection. Advanced network architectures have been devised to enhance detection accuracy, particularly for small-sized traffic signs. To effectively recognize traffic signs in intricate environments, numerous approaches have incorporated image segmentation techniques.

Additionally, attention-based detection methods have emerged, employing attention modules to extract ROIs from input images and fine-tune features within complex backgrounds^5^. By leveraging these strategies, the accuracy of traffic signs and potholes recognition has been greatly improved while minimizing the occurrence of false alarms. However, one major challenge arises when attempting to deploy deep neural networks on moving platforms. The process of deploying these networks is often time-consuming and computationally intensive, making real-time traffic sign detection on movable devices a formidable task. Furthermore, the recognition of minor traffic signs and potholes in complex environments is a critical aspect of traffic sign detection systems. Researchers have dedicated substantial efforts to develop effective techniques that leverage deep learning, image segmentation, attention mechanisms, and compression methods to enhance the accuracy and efficiency of traffic sign recognition, particularly for small-sized signs.

The current scenario lacks a comprehensive model capable of effectively alerting concerned authorities and drivers about the condition of roads. In this work, we propose an innovative approach that utilizes image recognition and computer vision techniques for the identification of potholes and traffic signs. This endeavor holds immense industrial potential, particularly in the domains of Driver Assistance Systems and Intelligent Autonomous Vehicles^6^. The first step of a typical pothole and traffic sign identification technique involves locating the precise position of potholes and traffic sign regions. The second step is assigning suitable classifications to the detected potholes and traffic signals. In our study, we have developed a gradient-boosting cascade classifier specifically tailored to accurately locate potholes and traffic signs even on challenging road surfaces. Additionally, we employ a vision transformer to effectively identify and assign labels to the detected potholes and traffic signs. By combining these techniques, we aim to provide a robust and reliable system for comprehensive road analysis and safety enhancement.

Based on existing literature, conventional approaches to pothole detection face limitations in identifying water-filled potholes and those either illuminated or concealed by tree shadows. Similarly, current traffic sign identification algorithms lack the ability to recognize perspective-oriented traffic signs and exhibit reduced accuracy in detecting illuminated signs at night. This research aims to introduce a more efficient and distinctive approach, specifically tailored for challenging climatic and topographical conditions.

The main aim of this work is to develop a model that will detect potholes and traffic signs in challenging environmental conditions. The notable contributions of this research include:

- Detection of water-filled potholes and potholes affected by illumination and obscured by tree shadows.
- Recognition of perspective traffic signs and tiny traffic signs affected by illumination.
- A novel model capable of detecting both potholes and traffic signs under diverse conditions.

The rest of the paper is organized as follows: Section II illustrates a review of existing literature relevant to the research topic. Section III illustrates detailed information on how the research was conducted, including the research design, participants, materials, and procedures. Section IV illustrates a presentation of the findings obtained from the research and interpretation and analysis of the results. Section V discusses a summary of the main findings and their significance. Section VI illustrates a list of all the sources cited in the paper.

## Literature survey

### Pothole detection

A novel model for pothole detection utilising CNN and LSTM was developed by Varona et al.^7^, which reached 93% accuracy. Dhiman et al.^8^ proposed using deep learning and stereo-vision analysis to spot the potholes in the road. A novel CNN method proposed by Aparna et al.^9^ has achieved 97% accuracy in identifying potholes in road pavements. The pothole detection model was developed by Sawalakhe et al.^10^ for use on a single-board Raspberry Pi. This model acquires the image/audio, processes it using computer vision algorithms, and then pinpoints the location of any potholes in the road. At last, the GPS coordinates of the pothole will be sent to the relevant authorities so that they may take the necessary next steps. A location-aware convolutional neural network (CNN) model was proposed by Chen et al.^11^. It leaves out the global context in favour of localised analysis of discriminative areas in road photographs. There are two steps: first, find the potholes, and then, sort them into categories. The overall accuracy of this model is 95.2%. An Internet of Things (IoT) model dubbed "DeepBus" was proposed by Bansal et al.^12^ to locate road defects in India. Internet-of-Things sensors are used to track the locations of the craters in real time. Users and authorities everywhere may now see an interactive map showing the precise locations of all known potholes. Users and administrators alike will get alerts so they may take appropriate measures as soon as possible.

### Traffic sign detection

The YOLOv3 layer-based network pruning along with a patch-wise approach for recognising very small traffic signals was introduced by Rehman et al.^13^. In addition, an anchor box selection algorithm was presented to determine the optimal anchor set. It decreases the overall miss rate and the proportion of false positives. It was trained and evaluated using data derived from traffic signs in Germany and Sweden, where it scored best in terms of mean absolute precision. A traffic sign-detection model was proposed by Wang et al.^14^, and it uses a refinement classifier and a lightweight super-clad detector. It boosts processing speed by using spatial statistics. The model only has 6.49 million parameters, making it rather simple. It was tested and refined using the Tisunga—Tencent 100k dataset, where it achieved 92.16% mAP with a per-frame processing time of 0.150 s.

To better recognise traffic signs, Wang et al.^15^ suggested a lightweight approach based on YOLOv4 tiny. An enhanced K-means clustering technique is employed to increase the recall rate and preciseness of the target position. A large-scale feature map technique is presented to enhance the precision of large-scale tiny item detection. The mAp and recall rates were both increased by 5.73 percentage points and 7.29 percentage points, respectively, after being trained and evaluated on the TT 100k dataset. To detect and identify traffic signs, Cao et al.^16^ proposed an improved sparse R CNN model. To distinguish even the tiniest of traffic signals, a unique detection model is provided, and a multiscale fusion structure technique is used. All of the research relies on the TT100k dataset, which achieved 62.3 mAP.

The IFA-FPN method was developed for traffic sign recognition by Tang et al.^17^. The Tsinghua-Tencent 100k dataset (TT100k), the Swedish Traffic Sign Dataset (STSD), and the German Traffic Sign Detection Benchmark (GTSDB) are utilised for the evaluations. The experimental results demonstrate the efficacy of the proposed IFA-FPN in detecting traffic signs. When the recommended IFA-FPN is applied to the Cascade RCNN, it receives a mAP of 80.3% in GTSDB, which is 9.9% higher than FPN; a mAP of 65.2% in STSD, which is 3.5% higher than FPN; and a mAP of 93.6% in TT100k, which is 1.6% higher than FPN.

Satti et al.^18^ presented a system for recognizing traffic signs on Indian highways. The traffic sign objects are found using a cascade classifier, and the traffic signs are classified using CNN. It simplifies the model since CNN just takes the frames that include the traffic sign objects. The trials were conducted using the ICTS dataset, and greater mAP values were obtained. A technique for identifying traffic signs was presented by Yang et al.^19^. The colour probability model and colour HOG are used for feature extraction and localization. CNN is in charge of traffic sign categorization. The experiments are carried out and assessed using datasets from the GTSDB and CTSD.

Most existing methods exhibit low detection accuracy in low-light conditions, and they often struggle to detect occluded and perspective traffic signs. Additionally, many models lack the capability to identify traffic signs in low-light situations. Furthermore, there is a gap in addressing the detection of potholes covered by tree shadows and filled with water. Moreover, the size and severity of potholes are not quantified or adequately addressed in the current approaches.

In an effort to maintain focus on the road while driving, drivers often overlook traffic signs and potholes. Such lapses pose potential dangers to both the driver and surrounding individuals. This issue might be mitigated with an effective means of alerting the driver without requiring a shift in their attention. The majority of tasks related to traffic sign recognition and pothole detection have primarily been executed on foreign roads, where the road conditions significantly differ from those in India. This research proposal centers on the development of models specifically tailored for detecting traffic signs and potholes in Indian road conditions. The objective is not only to enhance road safety but also to instil a sense of confidence in drivers navigating unfamiliar or challenging routes.

Moreover, the application of AI with ML or DL techniques has revolutionized the world today. For instance, applications like predicting the air passenger traffic flow^30^, health monitoring in urban traffic in the VANET network^31^, tracking moving vehicles from the video footage for automatic traffic flow analysis^33^, object detection in video surveillance systems^34,35^, etc. Furthermore, In order to assist traffic flow analysis (TFA) and solutions that need the forecast of many traffic variables, such as driving behaviour, journey time, speed, density, incident, and traffic flow, the paper^32^ analyses the application of data fusion (DF) approaches in Intelligent Transportation Systems.

## Proposed system

### Overview

**Figure 1:**
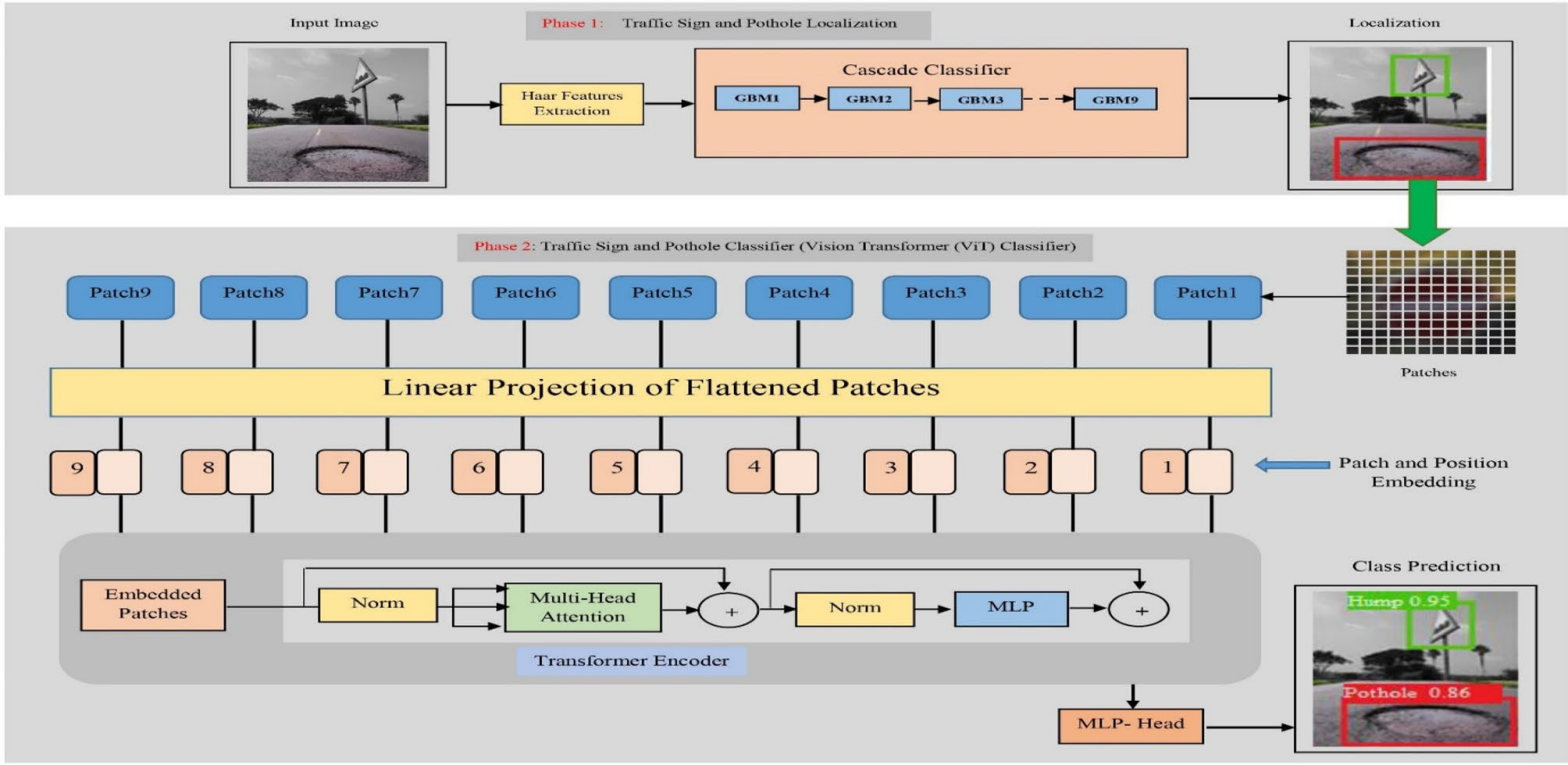
Proposed pothole and traffic sign prediction architecture.

Upon delving into the extensive literature survey outlined in Section II, it becomes apparent that the realm of recognizing potholes and traffic signs on Indian road surfaces has been largely unexplored. In light of this, the proposed system sets forth a groundbreaking approach to tackle this challenge and revolutionize the recognition process within the context of Indian road conditions. To begin with, a meticulously designed cascade classifier takes center stage, diligently working to pinpoint the exact locations of potholes and traffic signs strewn across the vast expanse of Indian roads. This initial phase serves as a crucial foundation for the subsequent steps. Subsequently, leveraging the power of Vision Transformer, a cutting-edge technique is employed to unleash the potential for precise prediction of both potholes and traffic signs. Figure 1 serves as a visual representation, showcasing the architecture of this innovative system, where the synergistic integration of cascade classifiers and vision transformers propels the realm of pothole and traffic sign prediction.

### Dataset

**Figure 2:**
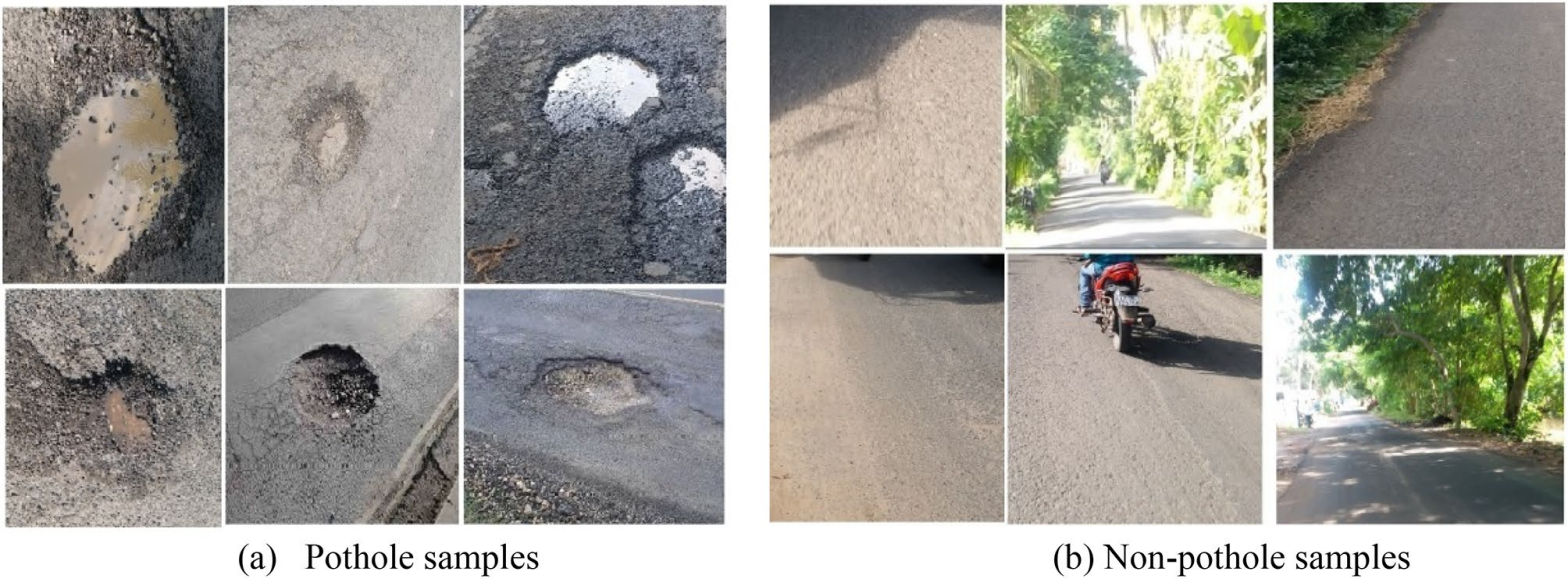
Pothole samples from dataset.

**Figure 3:**
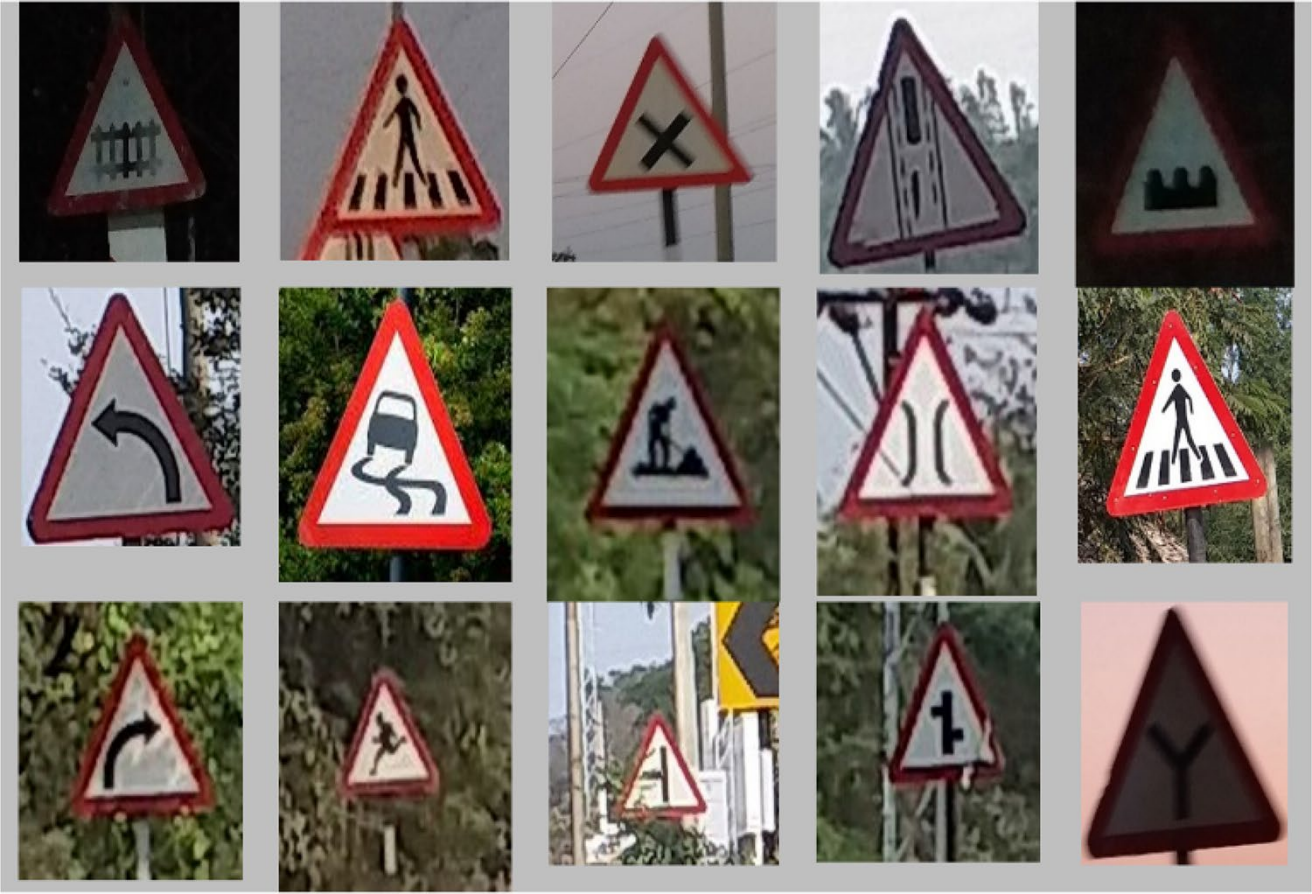
Collected traffic sign samples.

**Figure 4:**
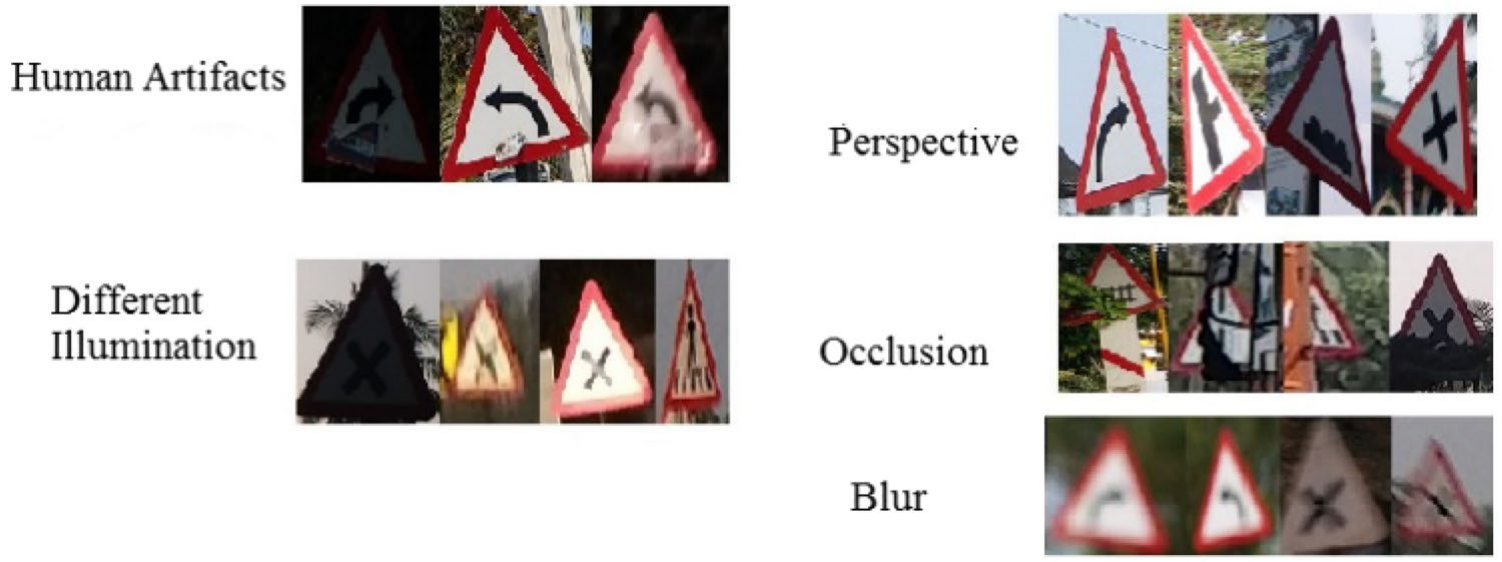
Challenging samples from the ICTS dataset.

To implement this model, 5000 pothole images and 19,775 traffic signs of 40 classes are acquired at diverse road conditions using the Samsung Galaxy C7 Pro mobile. The sample pothole and traffic sign images from the obtained dataset are shown in Figs. 2 and 3, respectively. Figure 4 shows the sample images of the challenged traffic signs for detection. Table 1 showcases the comprehensive distribution of the pothole and traffic sign dataset, meticulously curated to facilitate the training and testing of our innovative model. On the flip side, Table 2 shows a detailed breakdown of the dataset allocation specifically for both training and testing purposes. To ensure optimal image quality, a series of preprocessing techniques have been applied to the dataset. Notably, the implementation of Gaussian filtering and CLAHE^20^ techniques has been instrumental in enhancing the overall clarity and visual fidelity of the images, thus fortifying the foundation for accurate and reliable model training and evaluation.

**Table 1:** Distribution of collected potholes.

Environmental condition	Total images collected	Samples used for training (80%)	Samples used for validation (20%)
Day Time	*1700*	1360	340
Rainy	1600	1280	320
Low Light	1700	1360	340
Total	5000	4000	1000

**Table 2:** Distribution of collected traffic sign symbols^36^.

Labels	# of images	Training (80%)	Testing (20%)
200 m	508	406	81
100–500 m	272	218	44
Barrier ahead	288	230	46
Broad wideness ahead	421	337	67
Crossroad	523	418	84
Cattle	275	220	44
Cycle crossing	899	719	144
Dangerous dip	726	581	116
Falling rocks	200	160	32
Ferry	369	295	59
Gap in median	788	630	126
Guarded 200 m	274	219	44
Guarded 50–100 m	272	218	44
Hump	666	533	107
Left-hand curve	495	396	79
Left hair pin bend	510	408	82
Left reverse bend	653	522	104
Loose gravel	200	160	32
Major road ahead	775	620	124
Men at work	413	330	66
Narrow bridge	434	347	69
Narrow road ahead	400	320	64
Pedestrian	2033	1626	325
Right hair pin bend	739	591	118
Right reverse bend	235	188	38
Right-hand curve	912	730	146
Round about	120	96	19
Steep ascendant	912	730	146
Steep descendant	420	336	67
Staggered intersection	420	336	67
Slippery road	382	306	61
Side road right	382	306	61
Side road left	1350	1080	216
School ahead	288	230	46
Traffic signal ahead	170	136	27
Unguarded 200 m	382	306	61
Unguarded 50–100 m	272	218	44
Y-intersection	272	218	44
Total	19,775	15,819	3163

### Haar feature extractions

**Figure 5:**
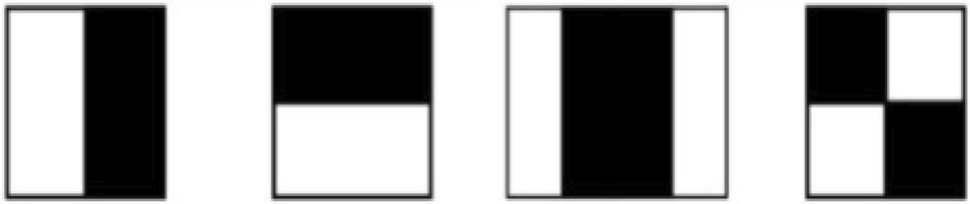
Convolutional Kernels used to extract Haar features.

In this section, we delve into the fascinating process of extracting Haar-like features to build a powerful cascade classifier. By applying the Convolutional kernels depicted in Fig. 5 to the image, we unlock a realm of valuable information. Kernels 1 and 2 skillfully capture the essence of edge features, while kernels 3 and 4 go a step further, to capture both edge and diagonal features. This strategic combination of kernels equips our cascade classifier with the ability to detect and distinguish these distinctive visual characteristics, ensuring reliable and precise identification.

### Object detection using cascade classifier

**Figure 6:**
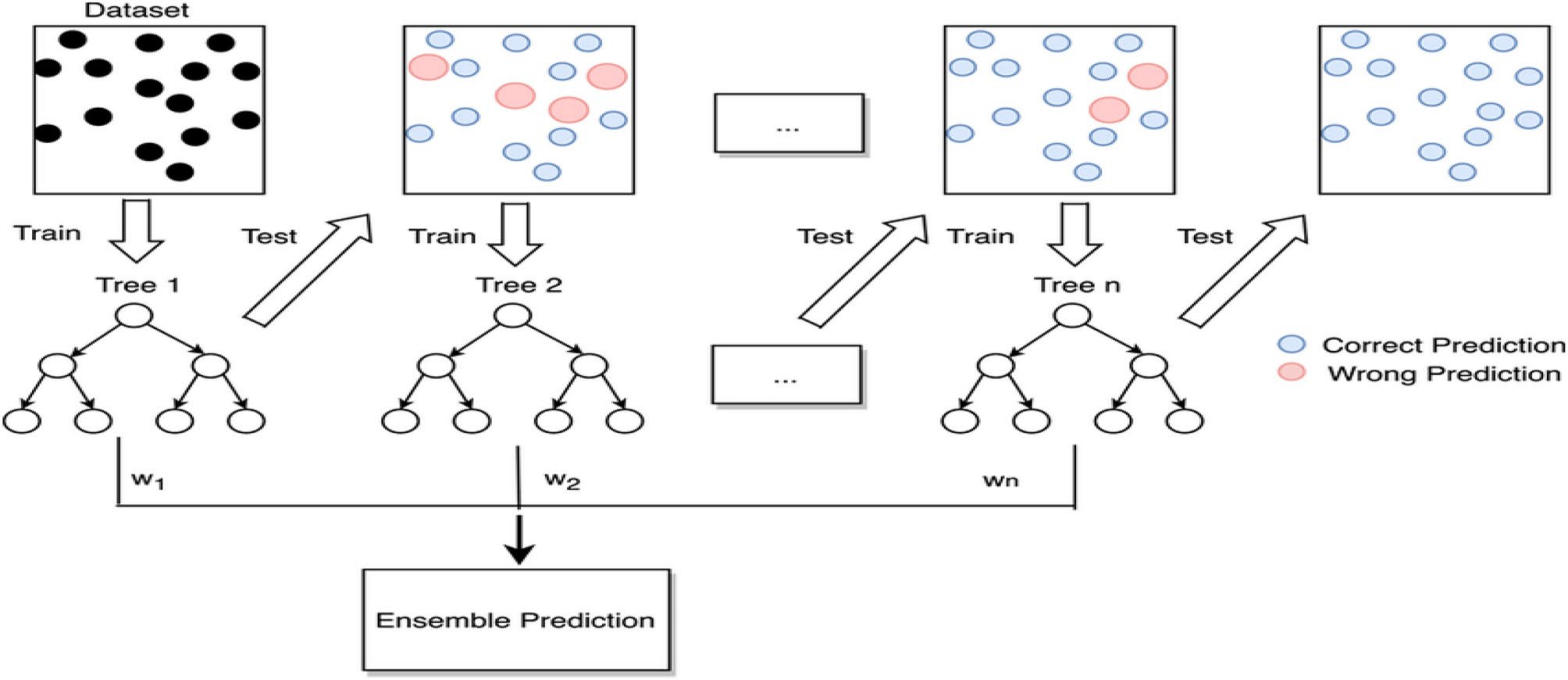
Training cascade classifier using gradient boosting method.

Boosting strategies, as a general approach, play a pivotal role in transforming weak learners from Section $C$ into robust learners. This technique empowers each newly constructed tree with an enhanced version of the original dataset. Initially, in the gradient boosting algorithm (gbm), a decision tree is trained, assigning equal weight to each observation. Following the evaluation of this primary tree, the weights of challenging-to-classify data points are increased, while the weights of easily classifiable observations are decreased. Consequently, the subsequent tree is constructed based on this weighted data, with the primary aim of improving the prediction accuracy of the initial tree. The fusion of the primary and secondary trees gives birth to a novel model. Next, the classification error of the two-tree cascade model is measured, and a new tree (third) is developed to predict the modified residuals. This iterative process is repeated for multiple epochs, wherein each successive tree aids in identifying observations that were not well-categorized by the previous tree models. The essence of gradient boosting lies in training numerous models incrementally, conservatively, and chronologically. By utilizing gradients in the loss function, gbm effectively identifies the areas where improvement is needed. The loss function serves as a guiding measure to assess the accuracy of the model coefficients. The selection and design of the loss function depend on the specific objectives and requirements of the problem at hand, dictating the conceptual perspective through which it is formulated and utilized. Figure 6 illustrates the process of training the cascade classifier.

The loss function is estimated based on the negative log probability that is converted to log.

*Step 1: Start the model with a constant*

1 $$F_{0} (x) = \arg \min \sum\nolimits_{i = 1}^{n} {L(Y_{i} ,\gamma )}$$

where $L$ indicates loss function and $Y_{i}$ and $\gamma$ represents observed and expected value respectively. At this level, the initial tree is to be constructed with a single leaf, after which trees with a larger depth are built. Usually, the mean $Y_{i}$ values are for regression and log values for classification.

*Step 2:* **for** m = 1 to M {M indicates no of trees to construct}

2 $${\text{(a)}}\;{\text{Compute}}\quad \gamma_{im} = - \left[ {\frac{{\partial L(Y_{i} ,F(x_{i} ))}}{{\partial F(x_{i} )}}} \right]_{F(x) = F(m - 1)(x)} \;for\;i = 1, \ldots n$$

Equation (2) returns the negative gradient of each observation for all trees that form the expected values of the previous classifier.

(b) Add a regression tree to the $\gamma_{im}$ values and generate terminal regions $R_{jm}$ for $j = 1, 2, \ldots m$.

3 $$(c)\;{\text{For}}\;j = 1, 2, \ldots m\;{\text{measure}}\;\gamma_{jm} = \arg \min \sum\nolimits_{{x_{i} \in R_{ij} }}^{{}} {L(Y_{i} ,F_{m - 1} (x_{i} ) + \gamma )}$$

Equation (3) gets the cumulative predicted values of each terminal node of all tees with shrink loss function, as well as the prediction of preceding learners.

4 $${\text{(d)}}\;{\text{Modernize}}\;F_{m} (x) = F_{m - 1} (x) + \eta \sum\nolimits_{j = 1}^{Jm} {\gamma_{jm} I(x \in R_{jm} )}$$

where $\eta$ is the learning rate.

*Step 3*: Get the output $F_{m} \left( x \right)$.

Thus, gbm constructs the ultimate prediction by accumulating inputs from each tree.

### Object classification using vision transformers

ViTs, short for Vision Transformers, revolutionize the field of computer vision by leveraging the Transformer model, originally designed for natural language processing. By adopting this powerful architecture, ViTs have garnered immense attention and have emerged as frontrunners in diverse image classification tasks, delivering state-of-the-art outcomes^21^. Figure 1 offers a comprehensive overview of Vision Transformers, illustrating their functionality. To process an input image, it is first partitioned into a grid of smaller regions known as patches. These patches typically encompass a fixed number of pixels, such as 16 × 16 or 32 × 32. Subsequently, the patches are transformed into flattened structures and undergo linear projection, producing lower-dimensional feature vectors referred to as patch embeddings. These embeddings effectively encapsulate local information from distinct areas of the image, enabling comprehensive analysis and understanding.

The embedded transformer receives a sequence of 1D token embedding as an input. In order to handle the 2D images, the original image $x$ is flattened into a series of reshaped 2D patches $x_{P}$.

5 $$x \in R^{H \times W \times C}$$

6 $$x_{p} \in R^{{N \times \left( {P^{2} \cdot \;C} \right)}}$$

Here, $H$ and $W$ indicate the height and width of the input image and $C$ indicates the channel of the input image (either 1 or 3). ($P, P$) represents the height and width of each patch, $N$ is the total obtained patches and it can be computed by (7).

7 $$N = \frac{H\;W}{{P^{2} }}$$

These patches are aid as the actual input for the transformer. Equation (8) is used to reshape the patches and project them into D dimensions. The outcome of the projection is referred to as the patch embeddings.

8 $$z_{0} = \left[ {X_{class} ;X_{p}^{1} E;X_{p}^{2} E; \ldots ;X_{p}^{N} E} \right] + E_{pos} \quad {\text{Where}},\;E \in R^{{\left( {P^{2} \cdot C} \right) \times D}} \quad E_{pos} \in R^{{\left( {N + 1} \right) \times D}}$$

Similar to the Transformer model in NLP, Vision Transformers incorporate positional information into the input data. Positional embeddings represent the spatial relationship between different patches in the image. They encode the position and order of the patches and are added to the patch embeddings. The patch embeddings, along with the positional embeddings, serve as input to a stack of Transformer encoder layers. Each encoder layer consists of two sub-layers: the multi-head self-attention mechanism and the feed-forward neural network.

The self-attention mechanism allows the model to capture dependencies and relationships between different patches. It computes weighted sums of the patch embeddings, where the weights are determined by the similarity between patches. The attention mechanism attends to all patches simultaneously, enabling global context understanding. After self-attention, a feed-forward neural network is applied to each patch independently. This network consists of fully connected layers, allowing non-linear transformations and feature extraction. The output of the Transformer encoder is a sequence of feature vectors, each representing a patch. To obtain a final classification, a global average pooling is applied to aggregate the patch representations into a single vector. This vector is then passed through a fully connected layer with softmax activation to produce class probabilities for different labels.

Training a Vision Transformer involves optimizing the model parameters, including the patch embeddings and the weights of the Transformer encoder, using labeled data. This is typically done through techniques like stochastic gradient descent (SGD) and backpropagation, where the model's predictions are compared to the ground truth labels, and the gradients are computed to update the parameters. During inference, a trained Vision Transformer can take an input image, extract patch embeddings, pass them through the Transformer encoder, and produce class probabilities for image classification tasks.

### The proposed algorithm

The following algorithm illustrates the procedure of the proposed method for traffic sign and pothole detection using cascade classifier with the vision transformer.

**Algorithm 1:**
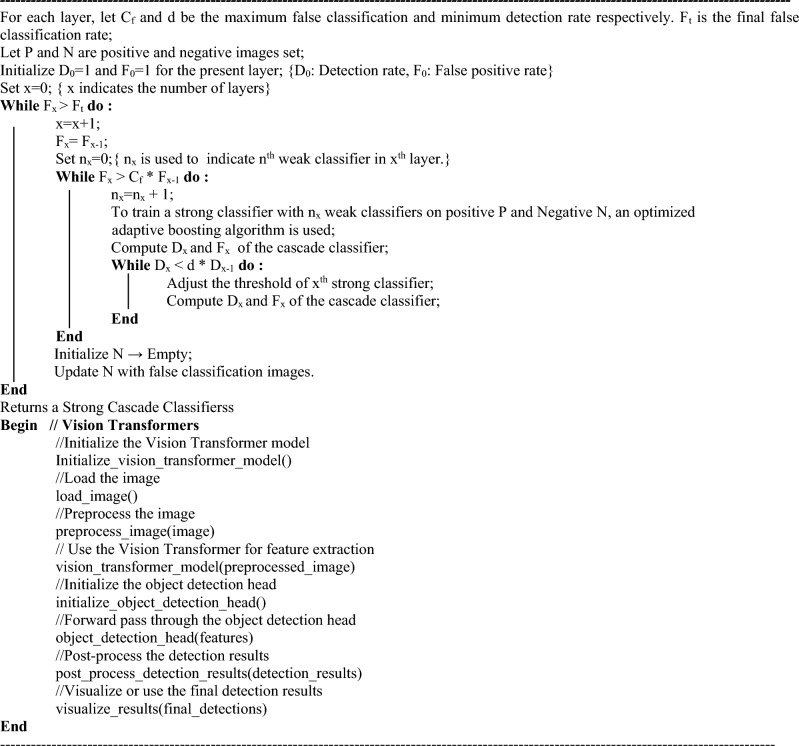
Traffic sign and pothole detection using cascade classifier with vision transformer

## Results and discussions

This experiment is conducted on the Windows 10 platform, featuring 64 GB of RAM, an 8 GB NVIDIA RTX 4000 GPU, and a 3.60 GHz processor. The proposed methodology is implemented using Python programming. The collected images of potholes and traffic signs are organized, as detailed in Tables 1 and 2, respectively. To train the model, 80% of the dataset is employed, while the remaining 20% is reserved for testing. The Cascade classifier ensemble, incorporating a sequence of nine gradient boosting techniques, is trained to detect pothole and traffic sign objects in road images, with bounding boxes delineating their locations. The parameters utilized for training the cascade classifier are outlined in Table 3. Subsequently, vision transformers are employed to predict the specific categories of the identified pothole and traffic sign objects. The parameters used for training the vision transformers are presented in Table 4. To appraise the efficacy of the proposed model, accuracy, recall, and Mean Average Precision (mAP) as performance/evaluation metrics are considered for the taken datasets. These metrics are then compared to those obtained from other cutting-edge techniques such as YOLOv3, YOLOv4, Faster RCNN, and SSD. This comparison is carried out to determine which system achieves the highest accuracy, recall, and mAP. This comparison aids in determining the most effective method for detecting potholes and traffic signs.

**Table 3:** Cascade classifier parameters.

Image size	Gradient boosting levels	Scale factor	Minimum neighbours	Max FAR	Minimum hit rate
64 × 64	9	2.0	4	0.27	0.997

**Table 4:** Vision transformer parameters.

Hyper parameter	Value
Learning_Rate	0.001
Weight_Decay	0.0001
Batch_Size	256
Num_Epochs	10
Image_Size	72
Patch_Size	6
Num_Patches	(image_size // patch_size) ** 2
Projection_Dim	64
Num_Heads	4
Transformer_Layers_Size	8

**Figure 7:**
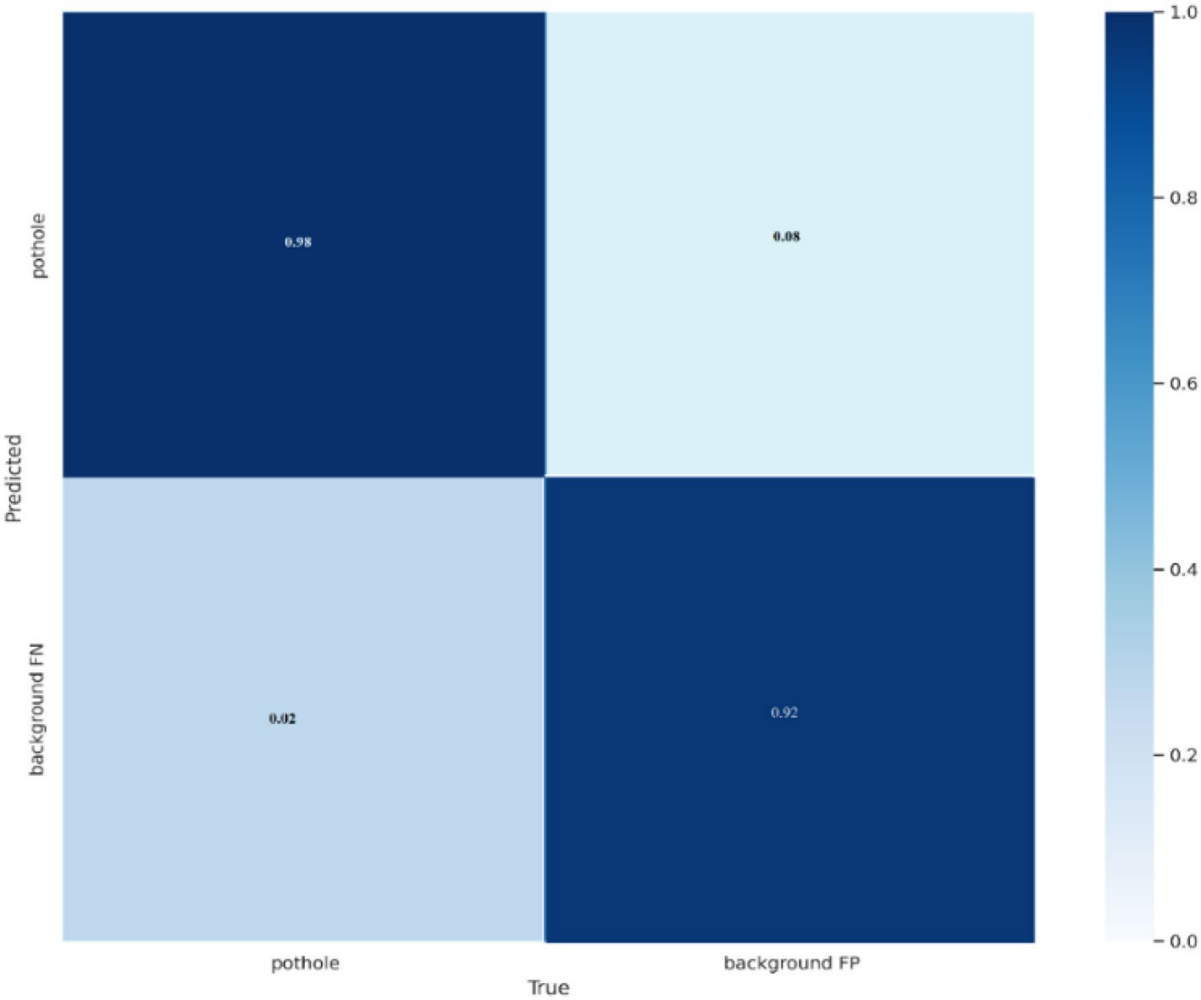
Confusion matrix of pothole prediction.

**Figure 8:**
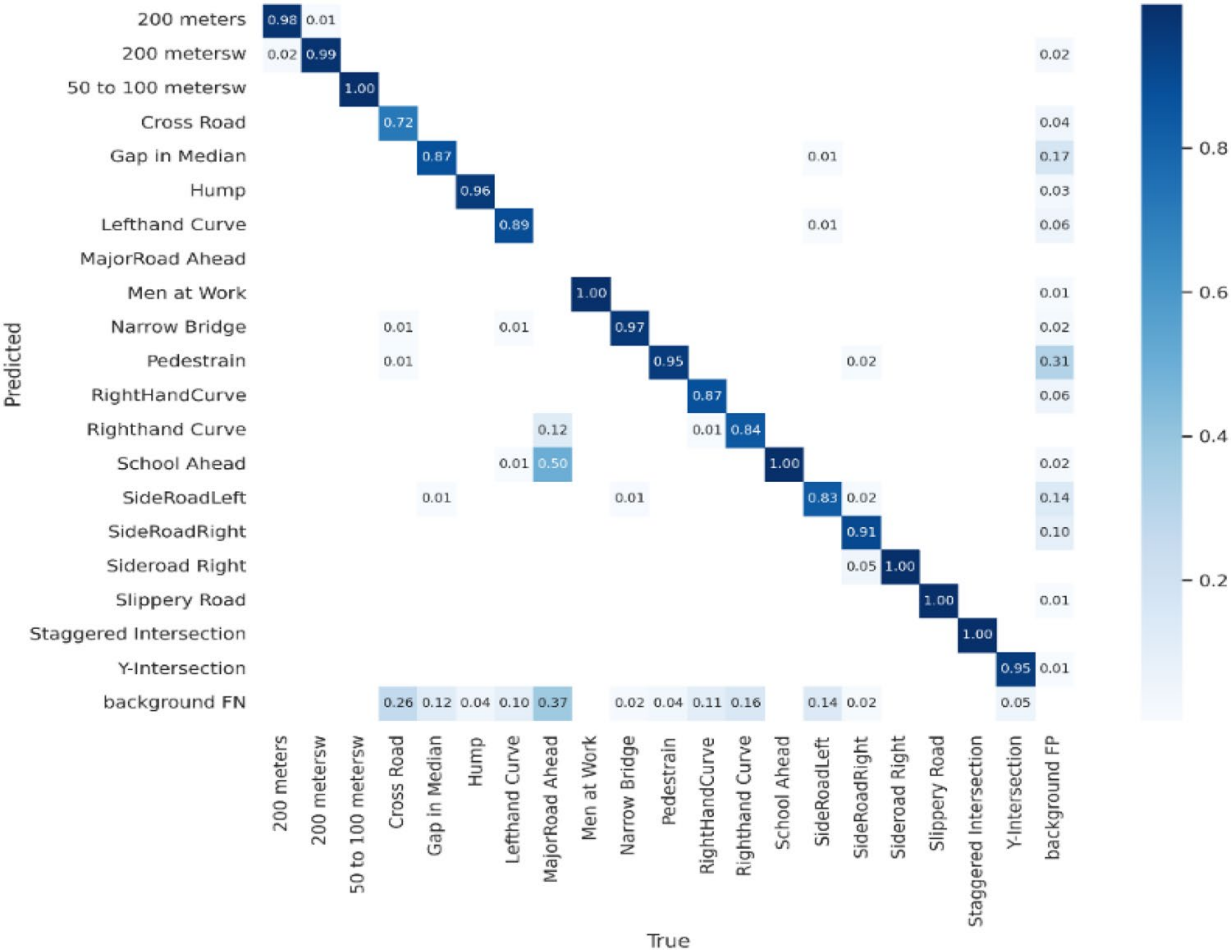
Confusion matrix of traffic sign prediction.

Figures 7 and 8 display the confusion matrices for pothole and traffic sign predictions, respectively. A confusion matrix is a performance measurement tool used in machine learning and classification tasks to evaluate the accuracy of a model. It's particularly useful when dealing with supervised learning algorithms where the output is categorical. The matrix itself is a table layout that allows visualization of the performance of an algorithm. It typically has four sections: *True Positive (TP), True Negative (TN), False Positive (FP) and False Negative (FN).* It is evident from Fig. 7 that the proposed strategy accurately predicted the potholes on the road. Likewise, Fig. 8 demonstrates the traffic sign prediction through the proposed model and notably, the TP rate is higher than the other three metrics. For both scenarios, the evaluation provides insights into the model's performance. Using these components, the confusion matrix provides a comprehensive view of how well a classification model performs by summarizing the model's predictions against the actual outcomes. It helps in understanding where the model is making mistakes, such as misclassifying one class as another.

**Figure 9:**
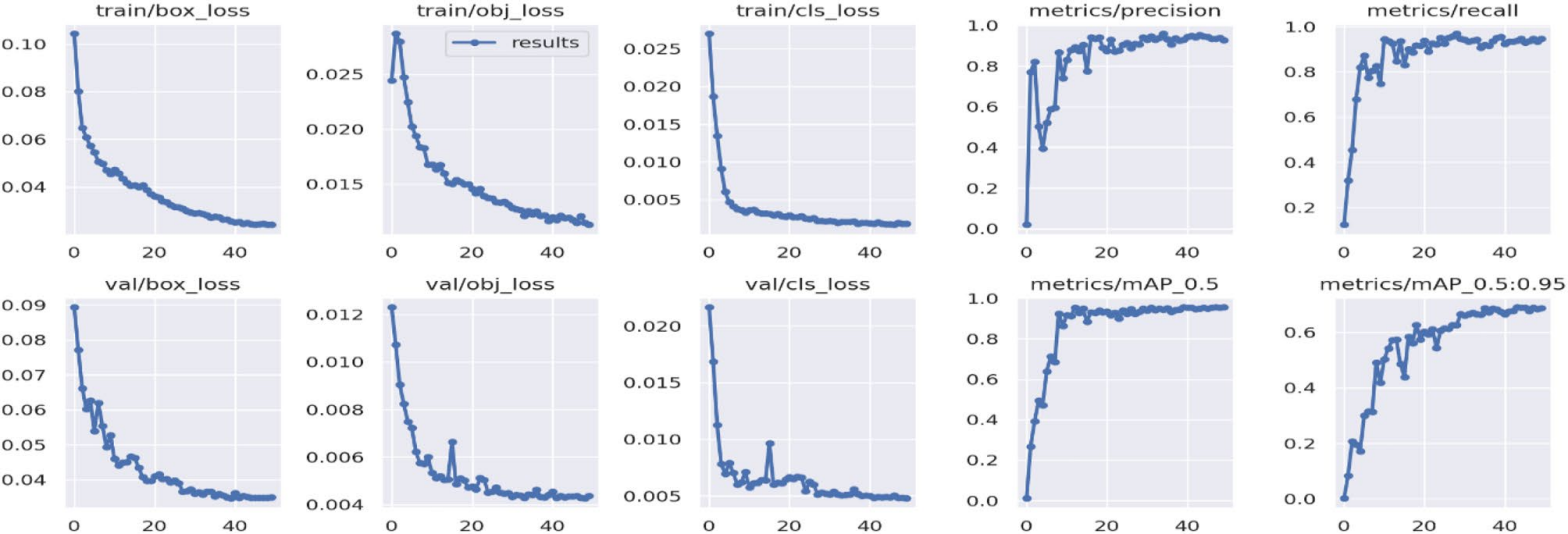
Precision, recall, and mean average precision (mAP) plots.

Figure 9 presents the Mean Average Precision (mAP) graphs generated during the model training. The evaluation of the trained model includes assessment using metrics such as precision, recall, and mAP. For instance, accuracy measures the overall correctness of predictions, while precision and recall focus on specific aspects like correctly identifying positive cases (presence of a sign or pothole). These metrics help in assessing the effectiveness of the models in identifying road signs or potholes, which is crucial for improving road safety and infrastructure maintenance.

**Figure 10:**
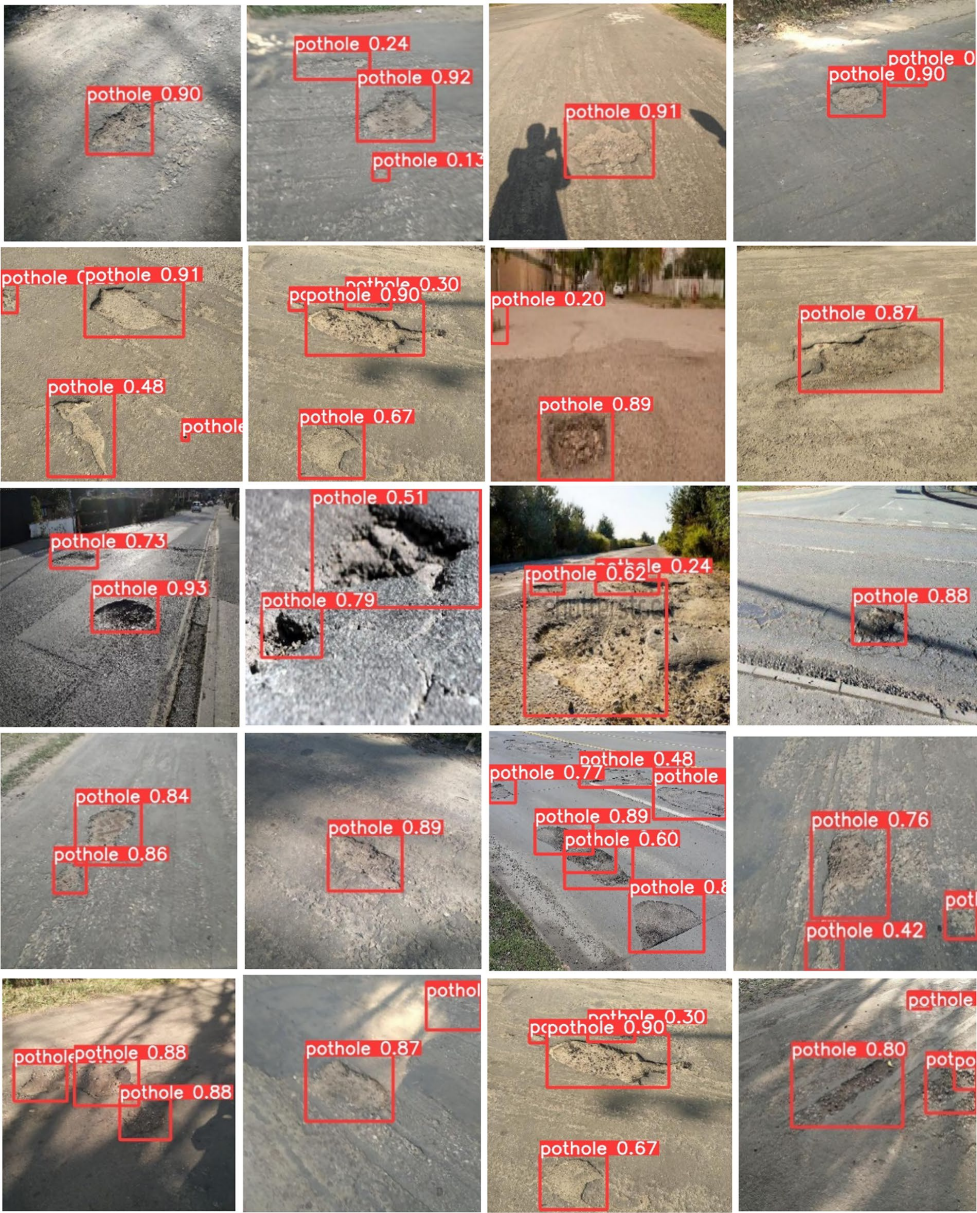
Predicting potholes in different conditions on the CCSAD dataset.

**Figure 11:**
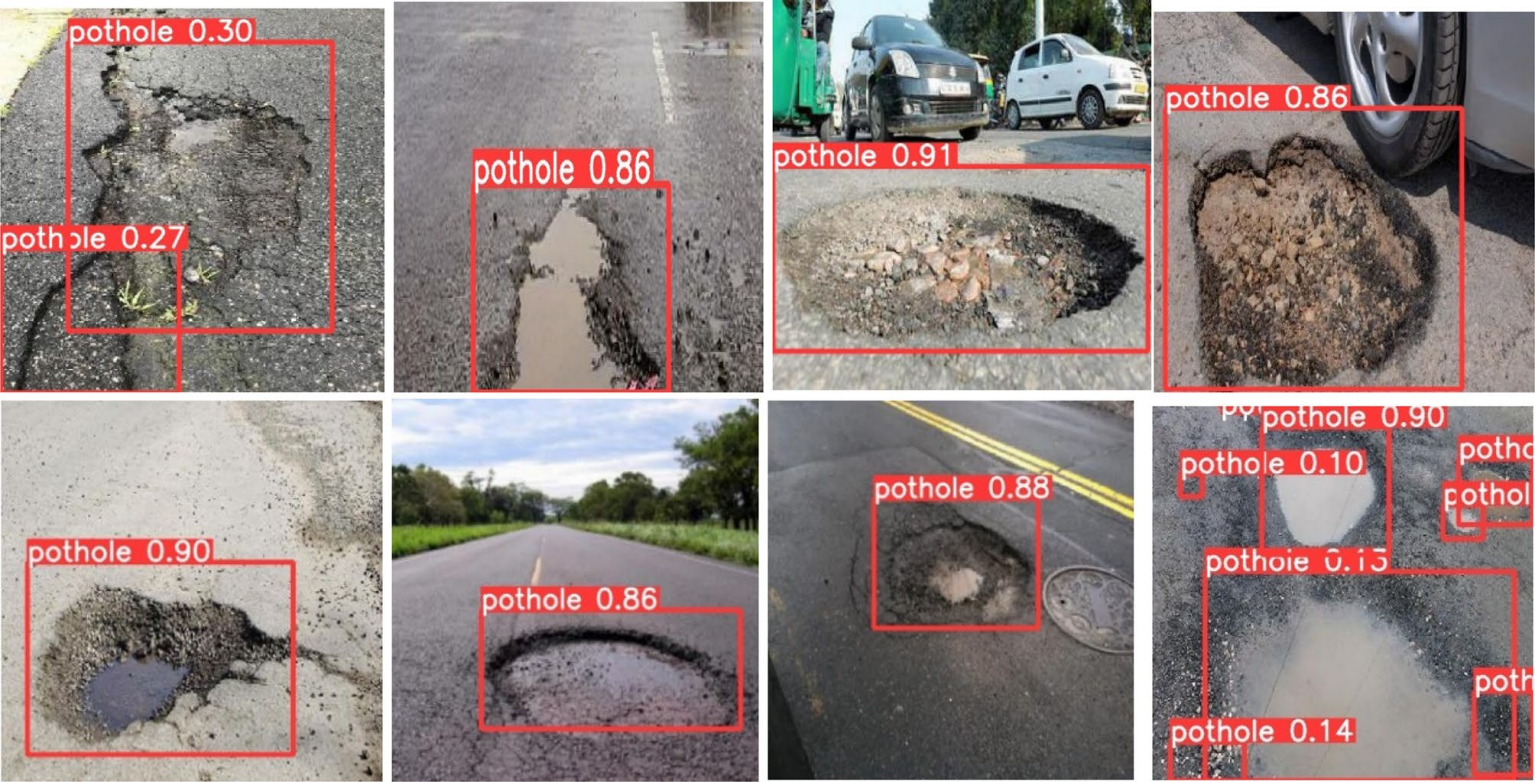
Predicting potholes filled with water.

**Figure 12:**
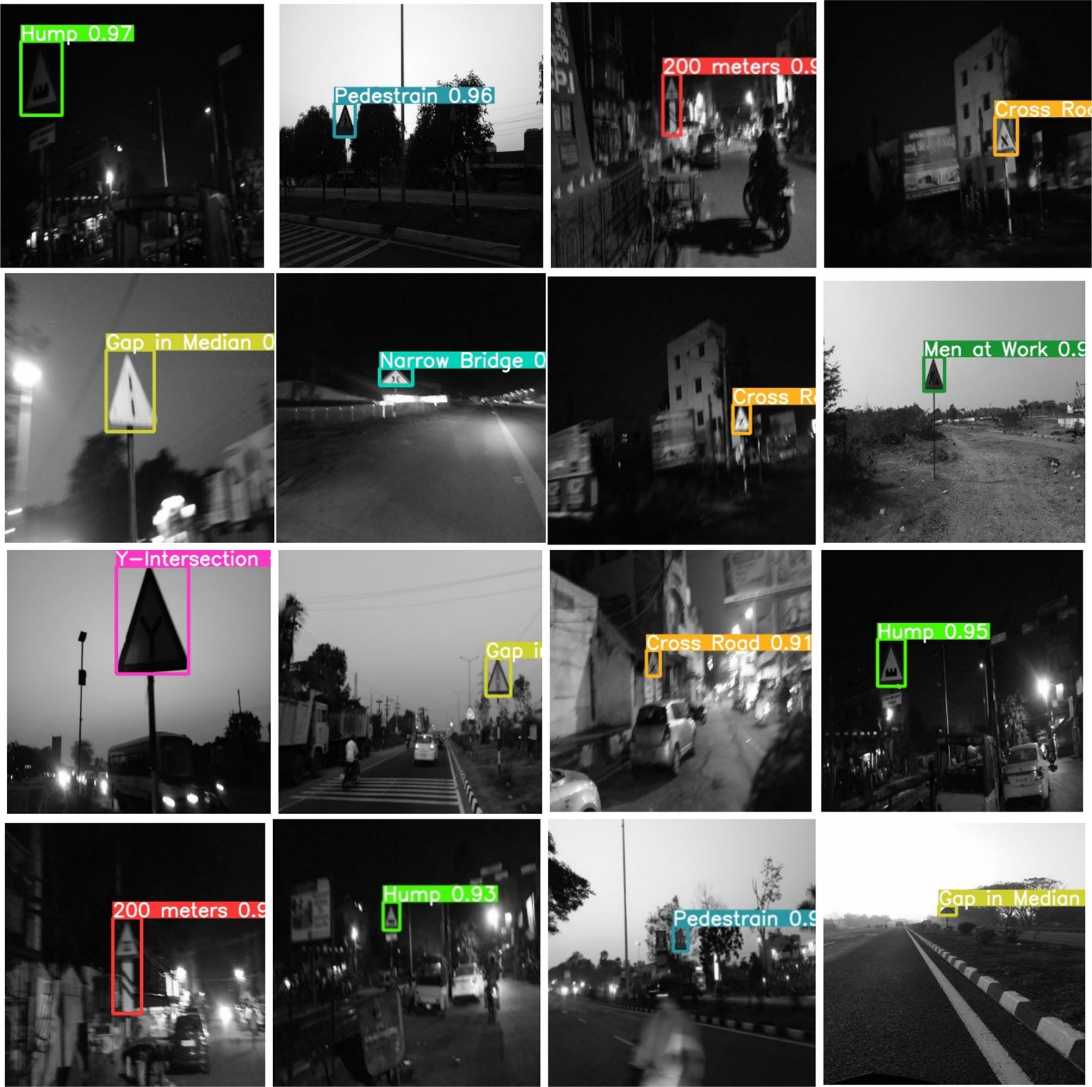
Predicting traffic signs at Illumination conditions.

**Figure 13:**
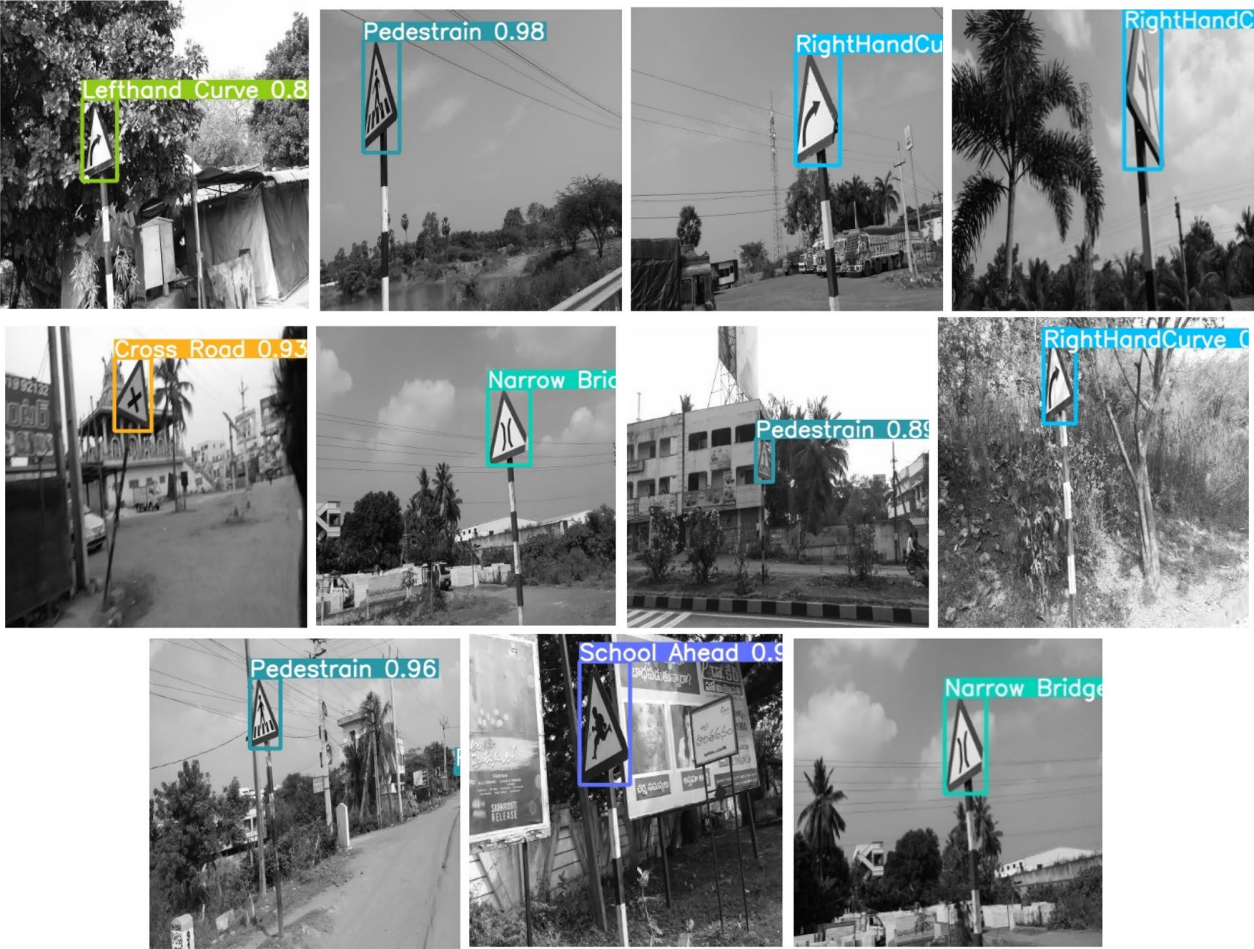
Predicting perspective traffic signs.

The proposed model is compared with other state-of-the-art techniques, including YOLOv3^22^, YOLOv4^23^, Faster RCNN^24^, and SSD^25^, demonstrating superior accuracy. Figure 10 showcases the comparative results on the ICTS^26^-based traffic sign dataset, while Fig. 11 provides a comparative analysis of the proposed model against other existing models on the GTSRB^27^-based traffic sign dataset. Additionally, Figs. 12 and 13 depict the comparative analysis of the proposed method with other object detection models on the KAGGLE^28^ and CCSAD^29^ datasets, respectively. It is apparent from these figures that the proposed approach outperforms existing strategies in terms of mAP, precision, and recall. The fusion of the cascade classifier with the vision transformer facilitates the inherent capabilities of the vision transformer in capturing global context, learning fine details, and adaptability to various conditions making them promising candidates for improving accuracy and robustness in traffic sign and pothole detection systems.

As depicted in Table 5, it is evident that the proposed methodology surpassed other methods, achieving a precision value of 97.90%, a recall of 95.69%, and a mean Average Precision of 97.14%. All methods highlighted in Table 5 underwent training and evaluation on the ICTS dataset. Moving on to Table 6, a comparative analysis of the proposed method on the GTSRDB benchmark dataset reveals that it achieved the highest detection accuracy among other object detection models, with a mean Average Precision of 98.57%. Tables 7 and 8 provide a comparative analysis of the proposed method with other state-of-the-art object detection models on the KAGGLE and CCSAD pothole datasets. Across both pothole datasets, the proposed model demonstrates superior detection performance, yielding a mean Average Precision of 97.27% and 97.17%, respectively.

**Table 5:** Comparative analysis on ICTS based dataset.

Methodology	Precision (%)	Recall (%)	mAP@0.5 (%)
YOLOv3	94.77	91.72	92.50
YOLOv4	95.35	94.64	95.07
Faster RCNN	93.73	92.46	93.35
SSD	95.52	94.79	95.89
Proposed model	97.90	95.69	97.14

**Table 6:** Comparative analysis of the GTSRB dataset.

Methodology	Precision (%)	Recall (%)	mAP@0.5 (%)
YOLOv3	93.47	93.26	95.57
YOLOv4	94.14	93.74	96.21
Faster RCNN	94.23	94.60	95.07
SSD	95.19	95.97	97.55
Proposed model	98.09	97.64	98.57

**Table 7:** Comparative analysis of the KAGGLE dataset.

Methodology	Precision (%)	Recall (%)	mAP@0.5 (%)
YOLOv3	96.42	97.27	96.38
YOLOv4	96.30	95.46	94.52
Faster RCNN	95.58	96.10	96.22
SSD	97.02	**97.52**	96.31
Proposed model	98.09	97.15	98.27

**Table 8:** Comparative analysis on the CCSAD dataset.

Methodology	Precision (%)	Recall (%)	mAP@0.5 (%)
YOLOv3	95.77	94.06	96.50
YOLOv4	97.35	96.27	94.07
Faster RCNN	95.73	95.64	96.25
SSD	95.52	95.57	95.19
Proposed model	98.23	95.69	97.17

Figure 10 shows the detection results of potholes under illumination and tree shadow conditions. All the potholes are detected with higher prediction scores and tiny potholes are also detected with the proposed methodology. Figure 11 depicts the detection results of the potholes filled with water (wet potholes). The proposed method detects all the water-filled potholes with the higher prediction score and with less training time.

Figure 12 illustrates the prediction outcomes for traffic signs under night time conditions. Notably, traffic signs such as hump, pedestrian, 200 m ahead, crossroad, gap in median, narrow bridge, crossroad, men at work, y-intersection, gap in median, crossroad, hump, 200 m ahead, hump, pedestrian, and a gap in median exhibit higher prediction accuracy under various illumination conditions. Moving to Fig. 13, the prediction results focus on perspective traffic signs. Existing methods falter in detecting these signs, but the proposed model excels in accurately identifying them.

The proposed approach successfully identifies both potholes and traffic signs, even in challenging conditions. In particular, water-filled potholes pose a detection challenge for Faster RCNN and SSD. Although YOLOv3 and YOLOv4 detect some of these water-filled potholes, they struggle with tiny or small ones. Potholes obscured by tree shadows are detected by Faster RCNN, SSD, YOLOv3, and YOLOv4, but with a high false detection rate. In contrast, the proposed method achieves comprehensive detection of all potholes with high accuracy and significantly reduces false positives.

Regarding images of perspective traffic signs, Faster RCNN, SSD, and YOLOv3 encounter challenges in detection. While YOLOv4 successfully identifies a limited number of slightly angled perspective images, the proposed method outperforms by precisely recognizing all perspective images with superior detection accuracy.

**Figure 14:**
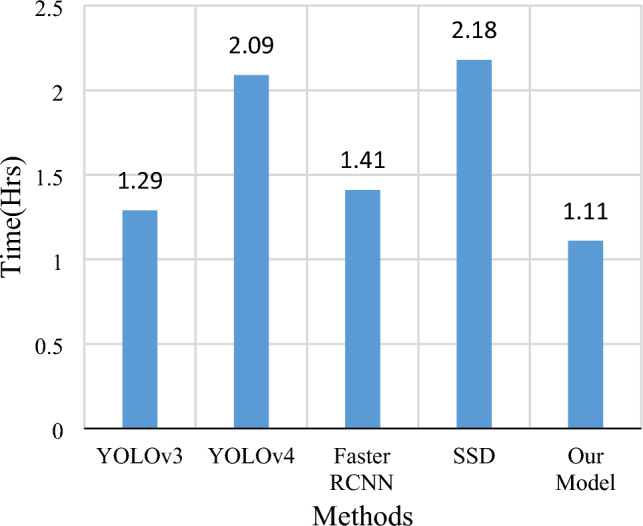
Training time comparison.

Figure 14 illustrates the comparative assessment of the proposed method concerning the training time required to train the model. The suggested model exhibits a shorter training duration in comparison to the other methods highlighted in Fig. 14.

This efficiency stems from the fact that the proposed model doesn't rely on image-specific biases, thanks to its utilization of multi-head self-attention. The model dissects images into a sequence of positional embedding patches, processed by the transformer encoder, thereby capturing both regional and global features of the image. Ultimately, when applied to datasets derived from ICTS, GTSRDB, KAGGLE, and CCSAD, the proposed model demonstrates superior accuracy with a reduced training time. The achieved mean Average Precision (mAP) of 97.14% for traffic sign detection and 98.27% for pothole detection surpasses benchmarks set by leading techniques such as YOLOv3, YOLOv4, Faster RCNN, and SSD, showcasing the effectiveness of the proposed methodology. Notably, our model showcases remarkable proficiency in accurately predicting potholes concealed in tree shadows, affected by varying illumination conditions, or filled with water, all achieved with a higher accuracy rate and reduced training time. Furthermore, our model exhibits exceptional competence in predicting traffic signs under challenging conditions like illumination variations, perspective distortions, and blurriness. The system's ability to recognize water-filled potholes and illuminated traffic signs, as well as handling perspective distortions and tree shading, marks a significant stride in improving safety under diverse environmental conditions.

## Conclusion

In this innovative study, we introduce a groundbreaking approach that transforms the landscape of predicting potholes and traffic signs on Indian roads. Our methodology initiates with the creation of a cascade classifier, adept at pinpointing the exact location of potholes and traffic sign objects, skillfully outlining bounding boxes around each identified entity. Taking a leap forward, we employ the state-of-the-art vision transformer to precisely forecast the specific class of potholes and traffic signs, pushing the boundaries of detection accuracy. Thorough training and evaluation of our model are conducted on prominent datasets, including ICTS, GTSRDB, KAGGLE, and CCSAD, utilizing quantitative metrics such as precision, recall, and mean Average Precision (mAP). Compared to state-of-the-art techniques like YOLOv3, YOLOv4, Faster RCNN, and SSD, the method achieves impressive recognition with a mAP of 97.14% for traffic sign detection and 98.27% for pothole detection.

The proposed model's performance in predicting potholes filled with water, navigating through challenges like obscured visibility due to tree shadows, or coping with changing lighting conditions represents a significant stride forward in road safety technology. Detecting water-filled potholes is particularly critical, as these can be highly hazardous and challenging to identify, especially when combined with shadows or varying light conditions. The proposed model's ability to discern these obscured or challenging instances substantially enhances road safety. Moreover, the system's remarkable proficiency in predicting traffic signs under adverse conditions like fluctuating lighting, perspective distortions, and blurriness significantly elevates its reliability in real-world scenarios. These conditions are commonly encountered on roads where factors like changing weather, time of day, or camera perspectives can impact the quality of visual data. Additionally, the model's adaptability to adjust to perspective distortions and identify traffic signs under tree shade enhances its versatility in handling diverse environmental scenarios. It has the potential to revolutionise automated systems' ability to perceive and respond effectively to various road conditions, thereby reducing accidents and contributing substantially to the overall efficiency and safety of transportation infrastructure.

Withstanding to aforementioned advantages, the combined cascade classifier and vision transformer system may pose computational challenges, especially when deployed in real-time applications on resource-constrained devices. Although the model is trained on diverse datasets, real-world variations may not be fully represented, necessitating continuous updates and expansion of the training datasets. Implement adaptive learning mechanisms to enable the system to continually improve and adapt to evolving road conditions and emerging challenges. Focus on optimizing the proposed approach for real-time implementation on edge devices, considering the computational limitations of such platforms. The study lays a strong foundation for advancing pothole and traffic sign detection, and ongoing research and development efforts can further refine and expand its applications in enhancing road safety.

## Data Availability

The datasets used and/or analysed during the current study are available from the author (Satish Kumar Satti) upon reasonable request.
